# Fraudulent Certificates from Germany

**Published:** 1886-11

**Authors:** 


### FRAUDULENT CERTIFICATES FROM GERMANY.

It is a too well-known fact that in the past many unworthy, uneducated men, in some cases without any knowledge whatever of our language, have come to this country in search of a dental diploma, in the hope, and too often the certainty, of being enabled, in a few months at furthest, to return with the diploma of a Doctor of Dental Surgery, and the colleagues of men of education and refinement. How they obtain the coveted degree from colleges that have always been considered respectable, is a mystery. It is possible that in some instances the College Faculties have been misled by fraudulent certificates furnished by this class of students. In some cases matriculation tickets at German Universities, which can be obtained by any one upon the payment of a small fee, have been accepted as certificates of attendance upon full courses of lectures, while the possessor of these worthless papers may never have attended for a day at the school at which he matriculated. Of late, attention has been called to an institution in Berlin, which has a high sounding name, and to the uninitiated may appear like a regular governmental educational school. If it really has any existence at all, it is nothing but a private school, and the names of none of its directors can be found in the list of regular German dentists. Its diplomas or certificates should not be recognized by any American Faculties. The name of the so-called college is as follows:

Erste Berliner Zahntechnische Schule, verbunden mit Policlinic fur Zahn und Mund-Krankheiten. Inhabor I. Malok.

